# Exploring the impact of COVID-19 on women’s alcohol use, mental health, and experiences of intimate partner violence in Wakiso, Uganda

**DOI:** 10.1371/journal.pone.0263827

**Published:** 2022-02-16

**Authors:** Amanda P. Miller, Stephen Mugamba, Robert M. Bulamba, Emmanuel Kyasanku, James Nkale, Fred Nalugoda, Gertrude Nakigozi, Godfrey Kigozi, Grace K. Nalwoga, Joseph Kagaayi, Stephen Watya, Jennifer A. Wagman

**Affiliations:** 1 Herbert Wertheim School of Public Health and Human Longevity Science, University of California, San Diego, La Jolla, California, United States of America; 2 Makerere University Walter Reed Project (MUWRP), Kampala, Uganda; 3 Africa Medical and Behavioral Sciences Organization (AMBSO), Uro Care Limited, Wakiso District, Hoima, Uganda; 4 Department of Community Health Sciences, Fielding School of Public Health, University of California, Los Angeles, Los Angeles, California, United States of America; University of the Witwatersrand, SOUTH AFRICA

## Abstract

**Introduction:**

Uganda confirmed its first COVID-19 case in March 2020, leading to country-wide closures and a stay-at-home order. Infectious disease pandemics can overwhelm adaptive coping capacity (e.g., general self-efficacy and resilience) and increase the risk for mental distress. For individuals experiencing intimate partner violence (IPV) and cohabitating with a perpetrator, stay-at-home orders can also increase risk of violence, which can further exacerbate mental distress. The present study explores women’s perceived self-efficacy and resilient coping, mental health outcomes (depression and COVID-19 related anxiety), hazardous alcohol use and IPV in the context of Uganda’s national 2020 lockdown.

**Methods:**

A phone-based survey was undertaken from June-August of 2020 in Wakiso District, Uganda. The study sample consisted of Africa Medical and Behavioral Sciences Organization (AMBSO) Population Health Surveillance (APHS) study participants who agreed to be contacted for future research. The analytic sample was restricted to women aged 13–80 years. Bivariate analysis and multivariable models explored associations between experiences of IPV and measures of adaptive coping, mental health and alcohol use.

**Results:**

A total of 556 women aged 13–79 years (mean age of 33.4 years) participated. Over half (55%) were currently married. The majority (60%) reported a decrease in alcohol use during the lockdown. Nearly half of the sample were experiencing physical or verbal IPV and reported an increase in violence during the lockdown. In adjusted analysis, alcohol use was associated with four times greater odds of recent physical IPV (aOR 4.06, 95% CI = 1.65–10.02, p = 0.0024), while participants had lower odds of experiencing any form of IPV as general self-efficacy increased (aOR 0.95, 95% CI = 0.91–0.99, p = 0.0308).

**Conclusion:**

Lockdown measures in Uganda may have mitigated increased alcohol consumption. IPV was exacerbated during lockdown; more than 2 in 5 IPV victims experienced increased physical or verbal violence. Development of programming and policies aimed at mitigating women’s risk of IPV during future lockdowns are needed.

## Introduction

Uganda confirmed its first COVID-19 case on March 21, 2020, leading to country-wide restrictions and closures, including suspension of public gatherings; school and non-essential business closures; discontinuation of public transportation; and enforcement of a national curfew [1]. In addition to the lockdown, several behavior change interventions were implemented to reduce transmission of COVID-19 in Uganda, including the placement of handwashing stations and alcohol-based hand rub dispensers in public places, and where possible, shifting to tele-working, tele-conferencing and the use of digital platforms for monetary transactions [2]. The enforcement of many COVID-19 lockdown measures, however, involved the use of excessive force by government forces and an armed community-policing paramilitary group called the Local Defense Unit (LDU) [3, 4]. The use of excessive force, under the guise of uniformly enforcing government directives for quarantine and curfew, ensured high rates of compliance with lockdown measures, leaving men, women and children confined in their homes for months. These measures were put in place to prevent a major public health crisis via infectious disease transmission; while protective in this regard, such measures also had the potential to exacerbate the sequelae of adverse health outcomes a global pandemic can cause, including negative mental health outcomes, [5, 6].

**Fig 1:**
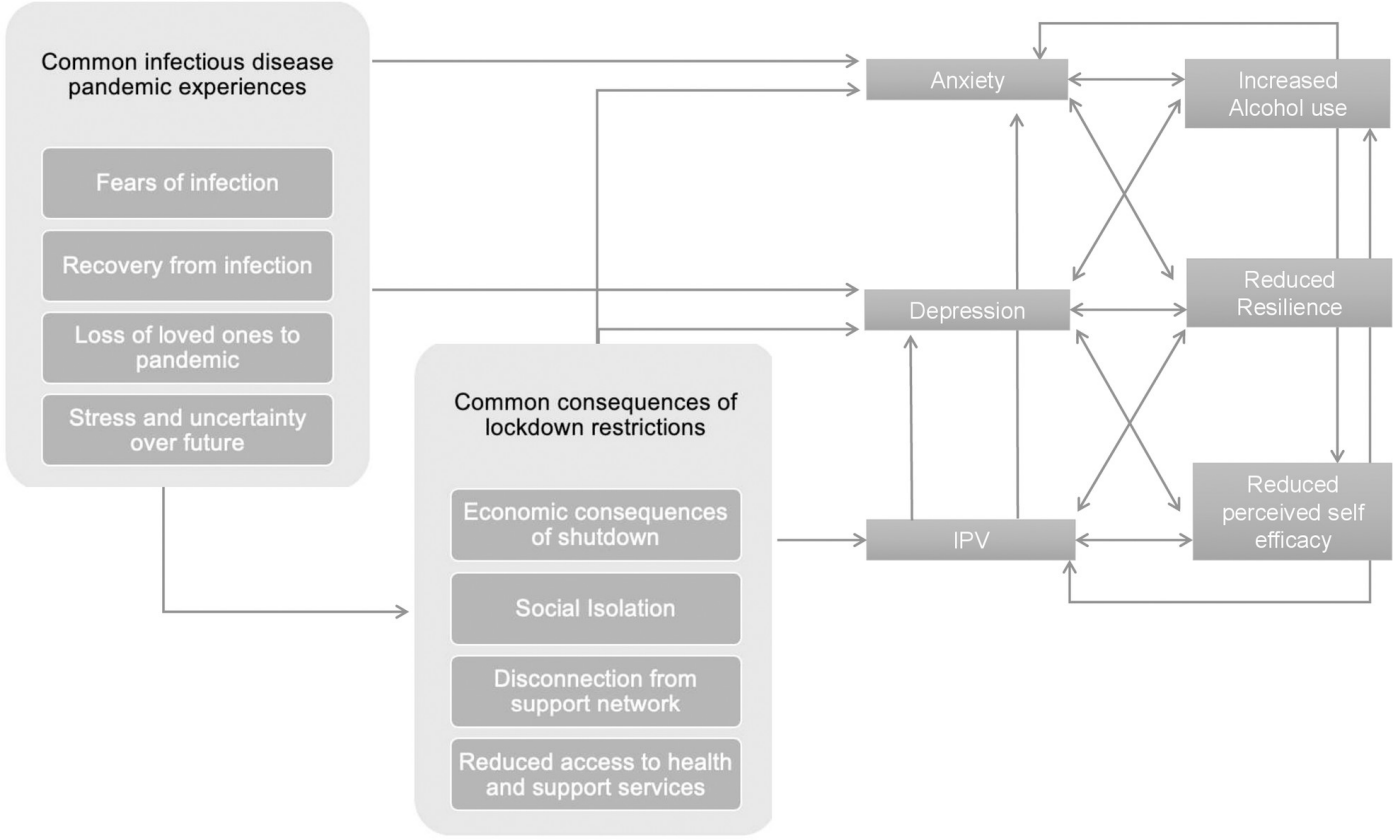
Conceptual model depicting pathways between an infectious disease pandemic, stay-at-home orders, mental health and behavioral sequelae.

Pandemics are massively disruptive to daily life, resulting in high levels of stress and increased risk for mental disorders and distress. Prolonged high stress situations, such as stay-at-home orders, can erode and fatigue levels of general self-efficacy and resilience and reduce access to adaptive coping strategies (e.g., exercise, meditation, seeking social support) [5, 7, 8]. Fig 1 presents a conceptual model depicting pathways between an infectious disease pandemic, stay-at-home orders, mental health and behavioral sequelae. Fear and worry regarding the unknown (from the infectious disease outbreak as well as the rippling impact on the economy and society), perceived health threats for one’s self and family, and adapting to the confines of life in quarantine can all lead to fear and anxiety, which has been documented globally [9–11]. A meta-analysis of 66 studies (n = 221,970) from the early stages of the COVID-19 pandemic and its impact on mental health found a pooled prevalence of 31.4%, 31.9% and 41.1% for depression, anxiety, and distress, respectively [10]. While increases in depression, anxiety and stress have been observed in the general population, some populations may be disproportionately affected including healthcare workers [11], persons infected with COVID-19 [12], persons who have lost a loved one to COVID-19, vulnerable populations at increased risk of severe illness [13], socially active individuals (who may be more affected by the isolation of lockdown) [14] and financially vulnerable individuals [15]. In low- and middle-income countries such as Uganda, where the majority of individuals earn their living in the informal sector (e.g., market vendors, owners of small business such as bars and bakeries where business and manufacturing cannot be carried out at home, agriculture, hospitality), working remotely and sustaining business operations during a lockdown is not feasible, and the accompanying stay-at-home order equated to financial uncertainty [16, 17].

The social and economic impact of quarantine for these individuals included loss of income, which could have psychological consequences such as experiencing increased anxiety around food insecurity and financial instability [18, 19]. A recent longitudinal study in South Africa, which has a substantial informal work sector and high rates of unemployment that were exacerbated by the lockdown, found that individuals who retained paid employment during the pandemic had lower depression scores, suggesting an enduring adverse effect of unemployment on mental health [20]. Fears of COVID-19 infection, stigma among those infected and isolation from peers can also adversely impact mental health [21]. Prior to the pandemic, depression was the second leading cause of disability adjusted life years in sub-Saharan Africa, representing a significant public health burden in Uganda [22]. Extensive global research has found depression to be associated with hazardous alcohol use [23–25], which refers to levels or patterns of drinking that increase risk for harmful (physical, mental or social) consequences for the drinker or other people [26, 27]. Hazardous alcohol use is common in Uganda [28] and may also have been exacerbated by circumstances related to the pandemic.

Evidence from prior pandemics, such as the 2003 SARS outbreak in Beijing, China suggests that individuals may increase their alcohol consumption as a coping mechanism for increased psychological and economic stress [29]. Early research from the COVID-19 pandemic suggests the same [30–32]. There is a well-established body of literature on alcohol use as a behavioral mechanism for coping with stressful or negative situations [33, 34]. Social Learning Theory posits that alcohol abuse is a form of “avoidance coping” in that it facilitates a person’s ‘‘removal” from experiencing or thinking about a stressful/negative situation [33, 35]. Avoidance coping (e.g., hazardous alcohol use) is considered maladaptive because it does not lead to a resolution (e.g., the elimination of stress). Furthermore, a large body of global evidence suggests that alcohol use, especially at hazardous levels, is also associated with intimate partner violence (IPV), another major public health issue in Uganda that may have been intensified by the lockdown [36, 37]. The World Health Organization defines IPV as any behavior within a current or former intimate relationship that causes harm to those in the relationship, including physical violence, sexual violence, emotional/psychological abuse and controlling behaviors. The terms ‘domestic violence’ and IPV are often used interchangeably [38]. In Uganda, qualitative and quantitative studies have linked alcohol use to IPV victimization among women and IPV perpetration among men. These studies indicate that alcohol use leads to violence that would not have happened if alcohol was not involved as well as increasing the severity of IPV [39–41].

Evidence from other recent infectious disease pandemics, such as the Ebola outbreaks in West Africa in 2014 and the Zika outbreak in the Dominican Republic in 2015, suggests that women and girls experience a greater burden from the disruption of mobility and isolation that occurs during pandemics [42, 43]. For example, for women cohabiting with their partner, stay-at-home orders increase the duration of time that victims of IPV are confined with their perpetrators each day and decrease their access to social support networks. Peru, another country with documented high rates of IPV prior to the pandemic, also enforced strict stay-at-home orders early in the COVID-19 pandemic; calls reporting domestic violence incidents increased by 48% from April to July 2020 [44]. In South Africa, where IPV is also prevalent, more than 87,000 gender-based violence complaints were filed within the first seven days of their mandated nationwide lockdown [45]. In Uganda, an increase in the rate and severity of IPV cases has also been reported during the COVID-19 pandemic and this increase has been attributed to women being isolated at home with their abusive partners [46, 47].

In addition to stay-at-home orders leading to increased violence through continual cohabitation between perpetrators and victims, the introduction of new pandemic-related stressors on the individual (e.g., fear and uncertainty), their social network (e.g., decreased access to support networks) and their household (e.g., loss of income), as well as maladaptive responses to such stressors (e.g., increased alcohol use) may increase the frequency and severity of IPV. Prior qualitative work in Uganda suggests that economic insecurity and concerns over finances are drivers of IPV in this setting [41, 48]. Patriarchal gender norms in Uganda uphold unequal power dynamics which are endorsed by both genders. Many men and women believe physical violence towards a wife is justified for disobedience [49] and traditional gender norms around masculinity dictate that men are expected to fulfill the role of provider (e.g., financially support and care for their families) [50]. The inability to fulfill this role can result in marital conflict [48]. Still, in many households, women are the sole breadwinners [4, 46, 51]; the low-paying informal sector is nearly entirely comprised of women, working in jobs such as market vendors, sex workers, hawkers (i.e., traveling/mobile salesperson), and caterers [51]. The closure of non-essential businesses and informal markets during the COVID-19 lockdown led to the loss of work for people in these jobs (many of whom are women) and severely affected their ability to earn income for themselves and their families, leading to economic hardship and food insecurity [17, 46, 52]. Regardless of the source of income, extant literature suggests that job loss, loss of household income and financial strain can serve as household stressors and potential catalysts for disputes and violence between partners [53]. This is true in families with and without a history of violence [53].

In Uganda, hazardous alcohol use (56.9% of alcohol users in Uganda have engaged in recent heavy drinking [28]), IPV (36% of women in Uganda have experienced physical IPV [54]) and depression (23.6% reporting depressive symptoms in the past 12 months [55]) were significant and interrelated public health issues prior to the pandemic [28], underscoring the importance of understanding if the context of the pandemic and subsequent lockdown exacerbated them. While the circumstances of an infectious disease pandemic place individuals at increased risk of several interrelated adverse health outcomes (as described above), individuals react to and handle stressful situations differently. Self-efficacy and resilience, which indicate an ability to adapt to changing and adverse situations, can serve as protective factors, mitigating the mental health impact of stressful life events such as a pandemic and its sequalae [56, 57]. Experiencing IPV may reduce perceived self-efficacy and increase avoidant/maladaptive coping (such as increased alcohol consumption) [58, 59]. Understanding how measures of coping (adaptive and maladaptive), mental health, and experiences of IPV were affected by COVID-19 can aid in the planning and preparation for future crises in Uganda and elsewhere. The present study examines women’s perceived self-efficacy and resilience, mental health outcomes (depression and COVID-19 related anxiety), hazardous alcohol use and IPV in the context of Uganda’s national 2020 COVID-19 lockdown. We also explore associations between experiences of IPV, mental health and coping measures.

## Materials and methods

### Study design and data collection

The present study uses cross-sectional data from a telephone survey implemented by the Africa Medical and Behavioral Sciences Organization (AMBSO) between June and August of 2020 as part of the AMBSO Population Health Surveillance (APHS) mixed-methods longitudinal cohort study, which has previously been described elsewhere [48]. The APHS is an open cohort study that began in 2018 and is currently in its third round of data collection. Briefly, APHS is conducted in six rural, urban, and semi-urban communities across the districts of Hoima and Wakiso in Uganda. The communities included in this study were selected through a community mapping exercise for their representativeness of the different community types that comprise Uganda. Data collection consists of a household census and enumeration of the community population, followed by a survey which covers a wide range of health topics and behaviors including sexual behaviors, healthcare utilization, food insecurity, nutrition, reproductive health, substance use, mental health and chronic diseases. Residents between the ages of 13–80 who provide informed consent (or assent for minors) are eligible to participate. At enrollment, individuals are assigned a participant ID (to link data across rounds) and provide consent to be contacted in the future for additional data collection opportunities. In addition to the survey, a blood sample for HIV testing and diagnosis is collected and pre- and post-test counseling services are offered. The data set from each round consists of responses from the household census and survey as well as results from biological specimen testing. Each survey round takes approximately 12–18 months to move through the six communities. The participation rate in the most recent round (round 2) was 57%. Data are collected in-person via same-sex one-on-one interviews, which are conducted by trained research assistants who are fluent in the local language of each community. Participants are compensated for their time and travel costs. In between the major survey rounds a mid-round telephone follow-up survey is conducted among those who participated in the previous major round (i.e., only those who participated in round two of the survey are eligible for the round two mid-round survey). The phone survey is also administered by same-sex trained research assistants. Typically, the mid-round survey reaches each community approximately six months after their participation in the major round.

The study sample consisted of persons who had participated in the APHS study between November 2019 and March 2020, when the country entered a national lockdown for the COVID-19 pandemic (which resulted in a pause of in-person data collection activities for round two). Given that data collection ended abruptly in the middle of a major round, only a subset of three APHS communities (the Wakiso cluster) were eligible for the mid-round phone survey; these three communities included one of each community type (rural, urban and semi-urban), making for a sample that is both representative of the larger APHS study population and Uganda more broadly. Individuals were eligible to participate if they had provided written informed consent to be contacted for follow-up data collection. Three attempts were made to reach each eligible participant. Oral consent to participant was secured (consent process was audio-recorded) from all phone survey participants. All surveys were administered to participants in the local language (Luganda). Data was collected on socio-demographics and health measures including depression, COVID-19 related anxiety, alcohol use, experiences of IPV and two measures of adaptive healthy coping (perceived self-efficacy and resilient coping) that were adapted to include items that addressed the lockdown. Participants were compensation for their time (5000 UGX, ~$1.42 USD); payment was provided via a mobile money transfer platform at the end of the interview using the participant’s preferred telephone number. Given our interest in understanding how the pandemic impacted women’s experiences of IPV, mental health, and healthy and unhealthy coping mechanisms, we restricted our analytic sample to female participants. This decision is further justified, given the overwhelming majority of IPV in the study setting (and globally) is perpetrated by males and experienced by females [49, 60]. The participation rate among women was 81%.

Seizing the opportunity to explore health outcomes in the context of the COVID-19 pandemic and national lockdown in Uganda in real time, the mid-round phone survey was adapted and used as an opportunity to ask study participants how COVID-19 was impacting various facets of their life, including their health. The study received Institutional Review Board (IRB) approval from the Clarke International University—Research Ethics Committee (CIU-REC) in Uganda (UG-REC-015, CIUREC/0059) and clearance from the Uganda National Council for Science and Technology. Updated clearance was received for all questions added to the phone survey as part of the COVID-19 module.

### Measures

#### Sociodemographic measures

Age was a self-reported measure: “how old are you in completed years?” Gender was a self-reported, dichotomous measure: male/female. To maximize confidentiality and safety of participants (data collection was not done in-person, precluding our ability to ensure privacy), and because lesbian, gay, bisexual and transgender (LGBT) rights are not protected and there is a general societal hostility toward sexual and gender minorities in Uganda—the survey did not assess if individuals were transgender or non-binary. Marital status was measured by a yes/no response to the question, “Are you currently married?” Community type was a three-level categorical variable: semi-urban, rural and urban. Educational attainment was measured using a categorical variable capturing the number of years of school an individual had completed. Responses were dichotomized into two categories: no schooling through primary school, secondary school through higher education.

#### Behavioral and psychological measures

The Alcohol Use Disorders Identification Test–Consumption (AUDIT-C) scale was used to measure hazardous drinking [61]. AUDIT-C is a modified version of the 10 question AUDIT instrument [62] and can reliably identify persons who are hazardous drinkers. The scale is comprised of three questions, each with five response options, scored 0–4. Participant responses are added together to calculate their overall score; the total possible score ranges from 0–12. In women, a cutoff of ≥3 is indicative of hazardous drinking [63]. Only participants that indicated alcohol use in the prior six months were asked the AUDIT-C.

The Patient Health Questionnaire (PHQ-9) depression measure was used to screen for depressive symptomology [64]. The scale is comprised of nine items; for each question, participants were asked how frequently they had experienced a given symptom in the past two weeks (not at all, several days, more than half the days, nearly every day). Participant responses across the items were added together to calculate their overall score. A categorical variable was also created based on previously established cut-offs [64]: no depressive symptomology (<5 points), mild depressive symptomology (5–9 points) and moderate to severe depressive symptomology (≥10 points). Only participants who provided responses to all nine items (i.e., did not respond “don’t know” or skip an item) had cumulative PHQ-9 scores calculated.

The Coronavirus Anxiety Scale (CAS) was used to measure participant COVID-19 related anxiety [65]. The scale is comprised of five items: (1) “I felt dizzy, lightheaded, or faint when I read or listened to news about the coronavirus”, (2) “I had trouble falling or staying asleep because I was thinking about the coronavirus”, (3) “I felt paralyzed or frozen when I thought about or was exposed to information about the coronavirus”, (4) “I lost interest in eating when I thought about or was exposed to information about the coronavirus”, (5) “I felt nauseous or had stomach problems when I thought about or was exposed to information about the coronavirus”. For each question, participants were asked how frequently they had experienced a given symptom in the past two weeks (not at all, rarely, several days, more than seven days, nearly every day). Participant responses were added together to calculate their overall score (which could range from 0–20). Using the optimum cut-off identified in the original validation study of this measure [65], a two-level categorical variable was also created to identify those with dysfunctional anxiety (cut-off score is ≥9). Briefly, anxiety is a natural functional response to perceived fear or danger that all humans experience. When that response is disproportionate to the risk posed and interferes with one’s ability to complete day to day activities (i.e., when the response reduces an individuals’ ability to function) then it can be considered dysfunctional.

Perceived self-efficacy was measured using an eleven-item scale [66, 67]. The first ten items were derived from the Generalized Self-Efficacy Scale [67]. The eleventh item was created specifically to capture pandemic-related perceived self-efficacy. Participant responses were added together to calculate their overall perceived self-efficacy score. The scale does not have a validated cut off; higher scores indicate higher levels of perceived self-efficacy. Psychometric assessment of the original ten item scale’s performance in 25 countries found a mean score of 29.55 (SD 5.32) [68].

Resilient coping was measured using a modified version of the Brief Resilient Coping Scale (BRCS) with a COVID-19 question added [66, 69]. Items included (1) “I look for creative ways to alter difficult situations”, (2) “Regardless of what happens to me, I believe I can control my reaction to it”, (3) “I believe I can grow in positive ways by dealing with difficult situations”, (4) “I actively look for ways to replace the losses I encounter in life”, (5) “I am coping well with the difficulty and stress caused by the COVID-19 pandemic”. Each item had five responses options: does not describe me at all, does not describe me, neutral, describes me and describes me well. Cronbach’s alpha for the five-item scale was checked, but responses to the original four-item scale were used to calculate participant resilient coping scores: low resilience (4–13 points), moderate resilience (14–16 points) and high resilience (17–20 points). Briefly, resilient coping can be thought of as the ability to adapt and respond to adverse circumstances, and reduce the negative impact of stressors through reframing, positive problem solving and offsetting losses [69]. Individuals in the high resilience category would therefore be the most equipped to buffer the effects of stressors and adverse circumstances.

Three types of IPV (verbal IPV, physical IPV and sexual IPV) were measured using 10 questions adapted from the Conflict Tactics Scales (CTS) [70], a globally validated measure utilized for IPV research. Participants were asked yes/no questions regarding their experiences of the three forms of IPV in the preceding six months, including, “In the past 6 months has your partner…”: Verbal IPV (1 item) “verbally abused or shouted at you?”; Physical IPV (6 items) “pushed, pulled, slapped or held you down?”, “punched you with fist or something that could hurt you?”, “kicked or dragged you, “tried “to strangle or burn you?”, “threatened you with a knife, gun, other weapon?”, “attacked you with knife, gun, other weapon?”; Sexual IPV (3 items) “used verbal threats to force you to have sex?”, “physically forced you to have sex?”, “coerced you to perform other sexual acts when you did not want to?” A yes response to any item in each IPV category indicated a yes for that form of IPV. To create the variable, “any recent IPV” responses across all three forms of IPV were collapsed, where a yes response to any of the 10 items indicated a yes response to “any recent IPV”.

Participants reporting alcohol use or IPV were also asked if they thought they had experienced changes regarding alcohol use or IPV as a result of the COVID-19 lockdown. Participants reporting any alcohol use in the past six months were asked, “How have your drinking behaviors changed during the COVID-19 pandemic? Would you say your alcohol use has increased, decreased or remained the same?” Response options included: “I have not drunk”, “I have drunk more”, “I have drunk less”, and my drinking behaviors remained the same”. Changes in IPV due to the pandemic were captured using a modified version of the Evidence-based Measures of Empowerment for Research on Gender Equity (EMERGE) IPV measure [71]. Participants who indicated they had experienced a specific form of IPV in the prior six months were asked “Do you believe that the "coronavirus/COVID-19"-lockdown made these things happen more or less often or remained the same?”

#### Analytic methods

SAS Studio software was used for analysis [72]. Sociodemographic and behavioral variables of interest were analyzed using descriptive statistics to characterize the analytic sample. Normality was assessed for continuous measures using the Shapiro-Wilk normality test. Descriptive analysis included frequencies for dichotomous and categorical variables, measures of central tendency for continuous outcomes, and stratified bivariate analysis of covariates by recent experience of any form of IPV and recent experience of physical IPV using χ^2^ analysis, fisher’s exact test (for variables with small cell sizes), two-sample T-test (normally distributed data) and Mann-Whitney U-test (when data violated the normality assumption). For continuous measures that were transformed into categorical variables using cut-offs identified from prior research (e.g. CAS and PHQ-9), when the distribution of the categorical variable in the overall sample resulted in small cell sizes (i.e., data did not support the analysis of this variable in bivariate or multivariable analysis), we reported the categorical variable for the overall sample but reverted to the continuous measure for additional analyses.

Adjusted analysis for associations between each of our mental health and coping variables and our two main IPV variables of interest (any IPV and physical IPV) were also explored in multivariable logistic regression models. The multivariable logistic regression models were adjusted for potential confounders that were identified a priori, including, age, employment, education, community type and marital status. Multicollinearity was assessed by examining intercorrelations between independent variables in the model as well as the tolerance and variance inflation factor (VIF). VIF and tolerance for all adjusted models and intercorrelations between independent variables suggested little multicollinearity between variables: all VIF approximated 1, tolerance values exceeded 0.1, and no correlations were >0.7. Internal reliability/consistency of included scales was measured by calculating Cronbach’s alpha coefficient.

## Results

Table 1 provides an overview of socio-demographic and behavioral characteristics of our study sample. A total of 556 women participated in the phone survey. Participant ages ranged from 13–79 years with a mean age of 33.4 years (SD = 13.7 years). More than half (54%) of participants resided in semi-urban communities and 55% were currently married at the time of the survey. Just under half (45.9%) of participants attained a secondary level of education or higher. The most common professions were agriculture and housework (33% and 30%, respectively).

**Table 1:** Descriptive characteristics of the study sample (n = 556).

Characteristic		n (%)
Age Mean (SD)		33.4 (SD 13.7)
Community Type		
	Semi-urban	301 (54.1%)
	Rural	204 (36.7%)
	Urban	51 (9.2%)
Educational Attainment		
	No Schooling/Primary	301 (54.1%)
	Secondary or above	255 (45.9%)
Primary Occupation		
	Agriculture	185 (33.3%)
	Housework	167 (30.0%)
	Trade/vending	87 (15.7%)
	Other	117 (21.0%)
Marital Status		
	Currently Married	308 (55.4%)
	Not Married	248 (44.6%)
Generalized Self-Efficacy Mean (SD)		36.0 (5.41)
COVID-19 Related Anxiety in past two weeks (n = 556)		
	No dysfunctional anxiety	550 (98.9%)
	Dysfunctional anxiety	6 (1.1%)
Resilient Coping (n = 556)		
	Low resilience	97 (17.5%)
	Medium resilience	187 (33.6%)
	High resilience	272 (48.9%)
Hazardous Alcohol Use in past two weeks (n = 256)		
	Non-hazardous drinking	252 (84%)
	Hazardous drinking	48 (16%)
Depression in past two weeks (n = 466)		
	No depression	424 (91%)
	Mild symptomology	33 (7.1%)
	Moderate/severe symptomology	9 (1.9%)
Verbal IPV in past 6 months (n = 379)		
	Yes	108 (28.5%)
	No	271 (71.5%)
Physical IPV in past 6 months (n = 379)		
	Yes	46 (12.1%)
	No	333 (87.9%)
Sexual IPV in past 6 months (n = 379)		
	Yes	28 (7.4%)
	No	351 (92.6%)
Any IPV in the past 6 months (n = 379)		
	Yes	116 (30.6%)
	No	263 (69.4%)

Participants’ self-efficacy scores suggested high levels of perceived self-efficacy and nearly half (48.9%) of participants fell into the “high resilience” category. Nine percent of participants reported some level of depressive symptomology and 16% had AUDIT-C scores indictive of hazardous alcohol use. Prevalence of verbal, physical and sexual IPV in the past six months was 28.5%, 12.1% and 7.4%, respectively.

Table 2 presents self-attributed changes in alcohol use and IPV frequency since the start of the COVID-19 pandemic among women who reported those experiences in the prior six months. When asked about their IPV experience(s) during the pandemic, more women attributed an increase in IPV victimization due to COVID-19 than a decrease. Among those reporting recent physical IPV, 47.2% reported an increase since the start of the pandemic, while 17.6% reported less violence and the rest reported no change. Among those reporting recent verbal IPV, 43.5% reported an increase since the start of the pandemic, while 15.2% reported less violence. Among those reporting recent sexual IPV, the majority (57.1%) reported no change due to the pandemic; 21% of women reported an increase and 21% reported a decrease in sexual IPV. Most women (60%) reported that their alcohol use had decreased since the pandemic started. Just over one-third (35%) reported their alcohol use had not changed and a small percentage (3%) reported an increase in alcohol use.

**Table 2:** Self-reported changes in alcohol use behaviors and frequency of IPV due to the COVID-19 lockdown.

Behavior	n (%)
Change in physical IPV among those experiencing IPV in past 6 months (n = 46)	
More often	20 (43.5%)
Less often	7 (15.2%)
The same	19 (41.3%)
Change in sexual IPV among those experiencing IPV in past 6 months (n = 28)	
More often	6 (21.4%)
Less often	6 (21.4%)
The same	16 (57.1%)
Change in verbal IPV among those experiencing IPV in past 6 months (n = 108)	
More often	51 (47.2%)
Less often	19 (17.6%)
The same	38 (35.2%)
Change in drinking behavior among those who drank in past 6 months (n = 231)	
Have not drank since lockdown started	3 (2.2%)
Have drank more	7 (3.0%)
Have drank less	87 (59.7%)
Drinking has remained the same	134 (35.1%)

Table 3 presents the standardized Cronbach’s alpha coefficients for the included scales. The cut off for an acceptable coefficient is typically 0.70 [73]. All scale measures had coefficients at or above this value except for the BRCS, which had a slightly lower coefficient value.

**Table 3:** Internal reliability estimates for included scales.

Scale	Number of Items	Standardized Cronbach’s Alpha Coefficient
BRCS	4	0.67
Modified COVID-19 BRCS	5	0.68
Generalized Self-Efficacy Scale	10	0.77
Modified COVID-19 Generalized Self-Efficacy Scale	11	0.79
PHQ-9	9	0.70
CAS	5	0.75

Table 4 presents the bivariate and multivariable analyses of associations between the mental health, resilient coping, and general self-efficacy measures and experiences of any IPV victimization in the past six months. Participants who had not experienced any IPV in the past six months had higher perceived self-efficacy than those who did report recent IPV (*Z = -*2.47, p = 0.0133). Those who had not experienced any IPV reported both lower mean depression scores than those who reported recent IPV (*Z* = 2.89; p = 0.0038) and lower mean COVID-19 related anxiety scores (*Z =* 6.19; p = 0.0129). In adjusted analysis, participants had lower odds of experiencing IPV as generalized self-efficacy increased (adjusted OR = 0.95; 95% CI = 0.91–0.99; p = 0.0308).

**Table 4:** Mental health, resilient coping, general self-efficacy, and hazardous alcohol use by experience of any IPV victimization in past 6 months (n = 379).

	Bivariate Analysis*				Multivariable Analysis for Odds of Experiencing Any IPV**	
** **	**Yes IPV (n = 116)**	**No IPV (n = 263)**	**Test statistic**	**p-value**	**OR (95% CI)**	**p-value**
Generalized self-efficacy Mean (SD)	35.3 (5.7)	36.9 (4.6)	*Z = -*2.47	0.0133	0.95 (0.91–0.99)	0.0308
COVID-19 related anxiety in past two weeks Mean (SD)	1.32 (1.9)	1.02 (2.24)	*Z =* 6.19	0.0129	1.04 (0.94–1.15)	0.4740
Resilience			*X*^2^ = 1.75	0.4166		0.5219
Low resilience	17 (14.7%)	34 (12.9%)			1.14 (0.58–2.27)	
Med. resilience	48 (41.4%)	94 (35.7%)			1.33 (0.82–2.17)	
High resilience	51 (44.0%)	135 (51.3%)			1	
Hazardous alcohol use in past two weeks (n = 218)			*X*^2^ = 1.98	0.1598		0.1490
Hazardous drinking	17 (22.4%)	21 (14.8%)			1.72 (0.82–3.62)	
Non-hazardous drinking	59 (77.6%)	121 (85.2%)			1	
Depression in past two weeks Mean (SD)	1.8 (2.3)	1.2 (2.2)	*Z* = 2.89	0.0038	1.11 (0.98–1.23)	0.0566

* Chi-squared, Fisher’s Exact Test, 2 Sample T-Test, Whitney-Mann U-Test.

**All models adjusted for age, marital status, employment, education level and community type.

Table 5 presents the bivariate and multivariable analyses of associations between the mental health, resilient coping, and general self-efficacy measures and experiences of physical IPV victimization in the past six months. In bivariate analysis, the only statistically significant differences observed were participant hazardous drinking and COVID-19 related anxiety The prevalence of hazardous drinking was higher among women reporting recent physical IPV victimization (37% vs 14%; *X*^*2*^ = 8.9; p = 0.0028). Participants who didn’t experience physical IPV in the past 6-months had CAS scores suggestive of lower levels of anxiety than participants who experienced IPV (*Z* = 2.63; p = 0.0084). In adjusted analysis, participants who consumed hazardous levels of alcohol had 4 times greater odds of experiencing recent physical IPV (adjusted OR = 4.06; 95% CI = 1.65–10.02; p = 0.0024).

**Table 5:** Mental health, resilient coping, general self-efficacy, and hazardous alcohol use by experience of physical IPV victimization in past 6 months (n = 379).

	Bivariate Analysis*				Multivariable Analysis for Odds of Experiencing Any IPV**	
	Yes IPV (n = 46)	No IPV (n = 333)	Test statistic	p-value	OR (95% CI)	p-value
Generalized self-efficacy Mean (SD)	35.1 (6.4)	36.6 (4.8)	*Z = -*1.24	0.2134	0.96 (0.90–1.02)	0.1692
COVID-19 related anxiety in past two weeks Mean (SD)	1.67 (2.2)	1.03 (2.1)	*Z =* 2.63	0.0084	1.10 (0.97–1.24)	0.1439
Resilience			*X*^2^ = 2.71	0.2576		0.3925
Low resilience	9 (19.6%)	42 (12.6%)			1.79 (0.73–4.40)	
Med. resilience	19 (41.3%)	123 (36.9%)			1.42 (0.70–2.87)	
High resilience	18 (39.1%)	168 (50.5%)			1	
Hazardous alcohol use in past two weeks (n = 218)			*X*^2^ = 8.94	0.0028		0.0024
Hazardous drinking	11 (36.7%)	27 (14.4%)			4.06 (1.65–10.02)	
Non-hazardous drinking	19 (63.3%)	161 (85.6%)			1	
Depression in past two weeks Mean (SD)	1.84 (2.2)	1.36 (2.3)	*Z* = 1.77	0.0764	1.09 (0.95–1.25)	0.2135

*Chi-squared, Fisher’s Exact Test, 2 Sample T-Test, Whitney-Mann U-Test.

**All models adjusted for age, marital status, employment, education level and community type.

## Discussion

This paper explored the prevalence of resilient coping, perceived self-efficacy, COVID-19 related anxiety, depression, hazardous alcohol use and experiences of IPV among Ugandan women in the context of the 2020 national COVID-19 lockdown in Uganda. A combination of previously validated and novel measures adapted specifically for this pandemic were used. Through provision of timely estimates of the prevalence of and associations between several pertinent interrelated health issues, this exploratory work has important policy implications.

We explored associations between mental health, resilience, self-efficacy, and hazardous alcohol use and two measures of IPV: any form of IPV (which included verbal, physical and sexual IPV) as well as experiences of physical IPV, specifically. While the associations between both measures of IPV and all covariates were in the expected direction (i.e., those experiencing IPV were more likely to have scores indicative of worse mental health and were less likely to have scores indicative of high levels of resilience and self-efficacy), there were differences in which exposure measures were significantly associated with each IPV outcome. Overall, participants demonstrated high levels of perceived self-efficacy and high to medium levels of resilience. We do not have other studies to compare the performance of the 11-item self-efficacy scale, but mean self-efficacy scores in our study sample on the original 10-item measure (data not shown) exceeded mean scores identified in a meta-analysis of 25 prior studies [68]. In adjusted analysis, women had lower odds of experiencing any IPV as general self-efficacy scores increased. Our analysis was cross-sectional so we cannot disentangle the temporal nature of this relationship (i.e., we cannot answer the question, “did women experience IPV, which subsequently lowered their perceived self-efficacy?”), but prior qualitative work with female IPV victims identified reduced self-efficacy related to experiencing IPV victimization [58]. Self-efficacy, which is closely related to the construct of empowerment, can play a critical role in a victim’s ability to effectively process and overcome trauma [74]. Self-efficacy has also been identified as a mediator between stressful life experiences and depression [75, 76]. Further, pathway analyses suggest the relationship between stressful life events, reduced self-efficacy and depression may be cyclical, with depression leading to increased vulnerability to future stressful events, which then further erodes self-efficacy and leads to subsequent increases in depression [75]. These studies highlight the importance of self-efficacy as a protective factor against mental health sequelae and the need to include programming and resources to improve and maintain perceived self-efficacy in public health pandemic planning efforts.

The observed relationship between hazardous alcohol use and IPV victimization in women in our sample is consistent with existing literature [36]. Women experiencing physical IPV were significantly more likely to consume hazardous amounts of alcohol compared to women reporting no physical IPV. Alcohol use by one or both partners is associated with increasing severity of IPV (including the escalation of arguments into physical violence) [77]. In a seven-country multi-site qualitative study (which included Uganda), the relationship between alcohol use and IPV was explored; participants felt that their risk of experiencing violence increases when victims consume hazardous levels of alcohol [78]. Several theories exist describing the causal pathway between alcohol use and IPV. The proximal effects model [79] suggests that alcohol use intoxication reduces one’s ability to de-escalate arguments, which can lead to an increase in the occurrence and severity of physical IPV (i.e., alcohol use by one or both partners can lead to verbal arguments that escalate into physical altercations) [80]. The self-medication model [81] suggests that alcohol use can also be used as a maladaptive coping mechanism for persons experiencing trauma (such as physical IPV) (16), which can lead to subsequent experiences of IPV and increased severity of alcohol use (17, 18). It is surprising that we did not observe a significant association between the “any IPV” variable (which included sexual, physical and verbal IPV) and hazardous alcohol use. It is possible that this is a product of the inclusion of verbal IPV. Alcohol use by a male partner has been associated with verbal IPV perpetration [82, 83], but there is a dearth of literature exploring associations between alcohol use and verbal IPV victimization [36]. Alcohol use is associated with increased aggression (i.e., violence escalating from a verbal to a physical altercation). Therefore, the inclusion of persons solely experiencing verbal IPV may mean that we included persons who were not under the influence of alcohol and therefore were able to avoid the escalation of their argument to a physical conflict. Additional qualitative research that explores how alcohol use relates to experiences of different forms of IPV, especially verbal IPV, in this setting could improve our understanding of this finding.

A robust body of literature links depression and anxiety to IPV victimization. Associations between our mental health measures (depression and COVID-19 related anxiety) and IPV were no longer significant after adjusting for covariates and approximated the null- another unexpected finding. A recent meta-analysis examining correlations between mental health and physical IPV perpetration and victimization found that anxiety and depression were strongly correlated with physical IPV victimization among women [84]. Another recent systematic review investigating associations between sexual and/or physical IPV and mental health outcomes among college students in 25 countries found that violence victimization was associated with five different mental health outcomes, including depression [85]. An important difference between the present study and prior work examining these associations is the anxiety measure we used. The CAS is focused specifically on COVID-19 related anxiety (as opposed to general anxiety), which may not directly capture anxiety related to experiences of IPV. The significant association between COVID-19 related anxiety and IPV observed in bivariate analyses may have been spurious, disappearing after adjustment for confounding. Our recent work assessing the psychometric properties of the Luganda translated PHQ-9 (which was also used in this study) suggests that although this measure had acceptable internal consistency and construct validity, research focused on the development of more culturally relevant items to measure constructs of depression in this setting are needed [86]. Estimates of the prevalence of depressive symptomology in the present study are much lower than recent estimates in this setting using the same measure (9% vs 29.2%); eligible persons experiencing depression or COVID-19 related anxiety may have been less likely to agree to participate in the phone survey (non-response bias). Mental health is an under-researched topic in Uganda specifically, and sub-Saharan Africa more broadly. More work is needed to rigorously validate the performance of existing mental health measures and adapt and develop new measures for use in this context.

IPV is prevalent in Wakiso, Uganda; a previous round of the APHS found that 26.7% of women reported lifetime physical and/or sexual IPV [87]. In the present study, 12.1%, 7.4% and 28.5% of women reported recent (past six months) physical, sexual, and verbal IPV victimization, respectively. According to our data, verbal and physical violence were the most exacerbated by the lockdown and one-fifth of women experiencing sexual IPV (21.4%) reported an increase during this time period. There is a limited body of literature to which we can compare these findings, but our results are consistent with increases in IPV during COVID-19 lockdowns elsewhere. Both Peru and South Africa, which imposed strict lockdowns, experienced increases in reports of domestic violence [44, 45]. Chad, Senegal, and Mali all experienced increased reports of domestic violence as well (30%, 14% and 12%, respectively) [88]. A qualitative thematic analysis of online forum posts made globally during COVID-19 by IPV victims found increased incidence and severity of IPV was associated with several factors including: alcohol use, financial stress, being trapped with the perpetrator during lockdown and pre-existing health issues of the victim [89]. These findings collectively suggest that prevention and mitigation of IPV during lockdown—in addition to increased access to care and support services—should be a public health planning priority for future pandemics. Among those who reported a decrease in IPV during lockdown, qualitative work exploring which factors women felt drove this reduction and their household dynamics (such as whether both partners were in lockdown in the same physical space) could aid in the interpretation of these findings.

The African Union (AU) established guidelines for the creation and integration of gender responsive programming into COVID-19 national responses [90]. Recommendations from this report include increased allocation of special funds at the national and continental level to address violence against women and girls, establishing free hotlines for domestic violence reporting, IPV awareness campaigns, providing psychosocial support for women who experience violence, the establishment of emergency shelters and safe houses for victims of violence, the establishment of special policing units to address domestic violence during the pandemic and the establishment of special mechanisms to ensure timely prosecution of domestic violence cases. Initially, gender-based violence-related services were not designated as “essential” during the lockdown in Uganda, leading to disruptions in service provision. Uganda subsequently established a COVID-19 Essential Services Committee which has taken steps aligned with the AU recommendations; steps include the development of standard operating procedures (SOPs) to ensure continuity of gender-based violence, sexual and reproductive health services, and HIV services during the pandemic [88]. However, there is a dearth of published information available regarding the implementation of these SOPs and the utilization of services during subsequent pandemic-related lockdowns. We endorse the guidelines proposed by the AU and add two additional recommendations. Given the strong association between alcohol use and physical IPV victimization and the high prevalence of hazardous alcohol use in our sample, hazardous alcohol use (both as a stand-alone health issue and a risk factor for IPV) should be addressed in health promotion and awareness programming. We did not measure alcohol dependence in our sample, but it is probable that some individuals with hazardous alcohol use also have an alcohol use disorder. Resources for alcohol treatment (and detox) in this setting are scarce. However, given the ubiquity of smart phones and government-funded platforms (i.e., cost free and not requiring people to purchase data to use them) that could deliver mobile or video support services for those that wish to stop drinking could greatly increase access to social support—even during a stay-at-home order. Our second recommendation is that economic strain at the household level (a driver of IPV in this setting) must be mitigated through social assistance programs (e.g., cash transfer programs) that were adopted elsewhere in Africa in response to the pandemic [91].

One of the more unexpected findings from the analysis was the reported decrease in alcohol use during the lockdown. Sixteen percent of participants had AUDIT-C scores indicative of hazardous drinking in the prior six months. However, most participants (59.7%) who reported alcohol use in the prior six months indicated a reduction in drinking since the lockdown began. This contrasts with the increased alcohol use and binge drinking reported in studies conducted in the United States (US), another country that enforced a strict stay-at-home order [30, 32, 92, 93]. This decline in alcohol use could have been driven by two primary factors: reduced access to alcoholic beverages and the loss of income to purchase alcohol. In Uganda, drinking establishments closed as part of a presidential directive to stop the spread of COVID-19, making the acquisition of alcoholic beverages challenging. In addition, many individuals lost their jobs during the lockdown [94], resulting in reduced disposable income and in many cases, economic and food insecurity [95]. Under normal economic circumstances, in the rural communities of Wakiso, many individuals have only enough resources to meet their basic needs. To purchase alcohol during the lockdown, even if it was readily available, would have been challenging in a setting where nearly a quarter of people experienced food insecurity prior to the pandemic [87]. In contrast, the US experienced increased alcohol use during COVID-19; there, grocery stores (which sell alcohol in most states) remained open throughout the lockdown, and many states eased rules around restaurant sale of alcohol for offsite consumption and on demand delivery services (e.g., the phone app Drizly skyrocketed in popularity) [96, 97]. Many of these policy changes related to alcohol sales in the US have remained even though lockdown restrictions have lifted. Future studies will reveal if the impact of these changes on alcohol consumption patterns in the US are sustained outside of a national shutdown.

HabIT tracker, an online survey conducted in 83 countries, found an overall decrease in alcohol consumption among adults (≥18 years of age); however, it identified groups of individuals who were more vulnerable to increased alcohol use, including persons experiencing depression and anxiety, which they attributed to COVID-19 related stress [31]. Findings such as these could help explain the higher rates of hazardous drinking, as well as higher levels of anxiety, among women experiencing physical IPV—a stressor that may have been exacerbated by the pandemic. Increased alcohol use during the pandemic was a major public health concern among substance use researchers globally; concerns focused on increased consumption and untreated alcohol dependence, as well as alcohol-related sequalae such as IPV, suicide, and other mental health issues [98]. More work is needed to understand if specific vulnerable subgroups in Uganda, and sub-Saharan Africa more broadly, experienced increased alcohol use under these circumstances.

### Strengths and limitations

Some of the measures used in this study have not been previously validated in Uganda or a similar setting. The CAS had good internal consistency with a Cronbach’s alpha coefficient of 0.75. However, using the cutoff from the original validation paper (a score ≥9), we saw a very low prevalence of dysfunctional anxiety among women (1.1%). There is limited research on how anxiety is conceptualized and experienced in sub-Saharan Africa; while the measure demonstrated internal consistency, it may not have provided valid estimates for the prevalence of anxiety in our sample. The generalized self-efficacy scale demonstrated good internal consistency with and without the inclusion of the COVID-19 related item and we recommend its use in future work in this setting. While the BRCS had a sub-optimal Cronbach’s alpha score, both with and without the COVID-19 item (0.68 and 0.67, respectively; the traditional cut-off 0.70), it has been suggested that for exploratory research, a cut-off of 0.60 is acceptable [99]. Additional psychometric work is needed to optimize the performance of both the BRCS and the CAS measure in this setting, which could be achieved through a validation study in a larger sample using a survey that includes other measures of the same latent constructs (resilience and COVID-19 related anxiety). If item response theory methods [100, 101] suggest that specific items from these measures are not performing well, qualitative work could support the development of more appropriate scale items or novel measures in this setting. Alternatively, a less resource and time intensive approach than traditional qualitative methods, such as a rapid mixed-methods approach (e.g. free listing, card sorting, key informant interviews) could be applied to adapt these instruments [102].

The study had several other limitations. Although this is a longitudinal cohort study, the dataset is cross-sectional, precluding comparison of the prevalence of our variables of interest before and during the lockdown. Many of the variables explored in this analysis were added to the survey in response to COVID-19 and the lockdown; we therefore could not compare them with data collected prior to the pandemic. We did ask participants if they had experienced changes in alcohol use and experiences of IPV during the pandemic, but these responses were subject to recall bias. Furthermore, the questions relating to changes in alcohol use did not ask about changes to patterns of alcohol use (i.e., frequency and intensity of alcohol use), precluding detection of changes to/from hazardous drinking. This analysis was exploratory in nature and was undertaken to describe women’s perceived self-efficacy and resilience, mental health (depression and COVID-19 related anxiety), hazardous alcohol use and experiences of IPV in the context of the 2020 Uganda lockdown and generate hypotheses regarding the associations between perceived self-efficacy and resilience, mental health (depression and COVID-19 related anxiety), hazardous alcohol use and experiences of IPV. As such, we did not assess model fit as comprehensively explaining substantial variance in experience of physical and any IPV as it was not the goal of this paper. Therefore, adjusted models should be interpreted with caution. To ensure the health and safety of study participants and study staff, data collection occurred over the phone which may have impacted the reliability of our self-reported measures. Typically, APHS data is collected in-person in a private one-on-one setting. The survey contains personal questions of a sensitive nature and participant privacy in their own household could not be ensured. This may have resulted in underreporting of sensitive topics such as IPV, alcohol use and symptoms of depression and anxiety. Measures of undesirable behaviors (e.g., frequency of alcohol use) are subject to underreporting due to social desirability bias; having others present (in the household) during the APHS call may have amplified this bias, though attempts were made to minimize this risk by requesting that participants find private places to participate in the survey. Furthermore, a victim of IPV may have been unable to respond to the violence-related questions truthfully if the perpetrator or other family members were present at the time of the interview. This misclassification of persons by IPV status (i.e., persons underreporting experiences of IPV) may have also biased our estimated associations towards the null. Confounding by unmeasured variables (known and unknown) is another potential source of bias in this study. The conceptual model in Fig 1 highlights several potential confounders (e.g., economic and food insecurity, degree of social isolation) that may have biased our estimates. Therefore, results from this study (especially non-significant results), should be interpreted with caution. We also only report stratified analysis of physical IPV. We were also interested in exploring associations between sexual IPV and the mental health, alcohol use and coping measures, but too few women reported this form of IPV in the sample to support a stratified analysis. We were also unable to explore how sexual orientation and gender identity might have influenced our findings, given our study instruments did not assess whether participants were transgender or non-binary. Another potential source of bias is that the communities included in the survey were not randomly selected. However, communities included were chosen specifically for their representation of different community types in Uganda (to improve generalizability of findings), and all community residents between the ages of 13–80 who had participated in the prior round of the survey were eligible for participation—ensuring participants represented individuals from all strata of the study communities. Finally, the use of phones to recruit participants may have resulted in non-response bias; however, participation rates in our survey were high (81%). Despite these limitations, this analysis makes an important contribution to the evidence base by providing timely estimates of IPV, mental health outcomes, resilience, self-efficacy, and hazardous alcohol use in the context of an unprecedent public health crisis and endorsing policy recommendations.

### Conclusion

Future global pandemics are an inevitability. The present study suggests areas for additional work to prepare for the next global event. Situating our findings in the existing literature, the lockdown measures in Uganda, coupled with economic insecurity, may have mitigated increased alcohol consumption during lockdown in this setting. IPV was exacerbated by the lockdown with more than 1 in 5 IPV victims experiencing increased violence. Development of programming and policies aimed at mitigating the negative impact of lockdowns on women’s safety are needed. We endorse guidelines for the creation and integration of gender responsive programming into COVID-19 national responses established by the AU and provide additional suggestions. Qualitative work with victims around barriers and facilitators to accessing social support and other resources during the lockdown could inform the development and implementation of support services. Finally, validation efforts are needed to assess the validity of mental health and resilience measures used for the first time in this setting (e.g., CAS); psychometric assessment and appropriate adaptation of these measures is critical for their accuracy and use in the future.

## Supporting information

S1 Dataset (XLSX)
